# An institutional experience of a scripting‐driven automation of the emergent palliative radiotherapy workflow

**DOI:** 10.1002/acm2.70814

**Published:** 2026-09-23

**Authors:** Shadab Momin, Eduard Schreibmann, Justin Roper, Beth Ghavidel, Maxine Washington, Oluwatosin Kayode, Pretesh Patel, Ashish Patel, Hania Al‐Hallaq, Yulia Lyatskaya

**Affiliations:** ^1^ Department of Radiation Oncology Emory University Atlanta Georgia USA

**Keywords:** CBCT‐based radiation treatment planning, emergency palliative radiotherapy, simulation‐free workflow, treatment planning system scripting, workflow automation

## Abstract

**Background:**

Emergency palliative radiotherapy is often delivered under time‐constrained and high‐stress conditions, frequently bypassing conventional computed tomography (CT) simulation and relying on nonstandard workflows. These factors increase variability and the potential for error.

**Purpose:**

This work describes the development, implementation, and early clinical evaluation of a simulation‐free, cone‐beam CT (CBCT) based emergency radiotherapy workflow that leverages treatment planning system (TPS) scripting to standardize and automate deterministic steps while preserving clinical oversight.

**Methods:**

An ESAPI scripting‐driven emergency workflow was developed within the TPS to automate prescription handling, plan generation, beam configuration, dose calculation, and setup preparation using CBCT images. Safety checks were implemented in the form of prompts with the appropriate actions required. Alternatively, manual input was retained for image verification and treatment field borders definition. The workflow was implemented across multiple clinical sites within a single healthcare system and evaluated through dosimetric comparison of CT‐ and CBCT‐based planning. Metric parameters such as workflow time, reductions in manual interactions, and pre‐ and post‐training survey outcomes of automated 3D workflow were compared to the same parameters of the conventional 2D workflow.

**Results:**

The automated workflow successfully generated clinically acceptable emergency treatment plans with minimal manual input. CBCT‐based dose calculations demonstrated agreement with CT‐based planning within 2.5% when heterogeneity corrections were enabled and within 4.7% when disabled. Automation eliminated 33 manual data entry steps present in the 2D workflow and enabled treatment delivery with average on‐table time of approximately an hour. Survey results demonstrated strong staff support for automation, with over 90% anticipating improvement prior to training and 100% reporting perceived improvement following training. Staff comfort and confidence improved post‐training, and concerns regarding workflow clarity and safety decreased.

**Conclusions:**

A scripting‐based, simulation‐free emergency radiotherapy workflow was developed for safe and standardized palliative treatments while maintaining established clinical safeguards. By automating deterministic planning tasks and preserving human oversight for clinical decision‐making, the proposed approach improves consistency and staff confidence and has potential to improve safety and accuracy of palliative treatments.

## 1 INTRODUCTION

For oncologic emergencies such as spinal cord compression, superior vena cava syndrome, or life‐threatening bleeding, palliative radiotherapy (RT) has been shown to provide rapid symptom relief while limiting treatment‐related toxicity.^1^ The conventional RT workflow typically involves CT simulation, manual target delineation, treatment plan generation, and multiple quality assurance checks, a process which can span days. Although this timeline can be compressed to several hours for simple palliative plans, it may not be feasible for patients with severe pain or rapidly progressing symptoms.^2^ To address the time constraints associated with palliative RT, several simulation‐free approaches have been proposed and evaluated.^3^, ^4^, ^5^, ^6^

Historically, a two‐dimensional approach based on orthogonal kilovoltage (kV) or megavoltage (MV) imaging has been used for oncologic emergencies. While this workflow is more efficient compared to a standard RT workflow, it is error‐prone due to limited anatomical information and manual process. These challenges prompted investigations into utilizing 3D approaches for urgent radiation treatments for higher safety and accuracy of treatments while preserving time efficiency. One of the proposed strategies involves using pre‐existing diagnostic imaging, such as CT or MRI, to drive the RT planning workflow.^4^, ^6^, ^7^, ^8^ While diagnostic imaging–based approaches have the potential to reduce patient wait times and streamline care, they introduce challenges related to differences in patient positioning, couch geometry, and any immobilization that may be required. Additional uncertainties arise from differences in Hounsfield unit calibration between diagnostic and CT‐simulation scanners. Furthermore, patients requiring emergent palliation often experience rapid anatomic changes due to aggressive disease progression, potentially rendering previously acquired diagnostic imaging unsuitable for treatment planning. MRI–only workflows have also been explored in the palliative setting.^7^, ^9^ These approaches depend on the generation of synthetic CT images with accurate geometric and electron density information and typically require multimodality image registration for position verification. As a result, they may introduce additional complexity and uncertainty in emergent clinical scenarios. More recently, cone‐beam CT (CBCT)–guided RT has emerged as a practical and clinically viable alternative for urgent palliative treatments.^10^, ^11^, ^12^, ^13^, ^14^ This approach offers several advantages, including reduced susceptibility to anatomic changes, decreased reliance on additional hospital resources and staff, improved confidence in treatment geometry, and familiarity with the standard workflow. Advances in CBCT reconstruction and correction algorithms have further improved image quality and dosimetric accuracy, expanding the feasibility of CBCT‐based dose calculation for clinical use.^15^, ^16^, ^17^

Despite these technical advances, emergency RT workflows remain manually driven and inherently time‐constrained and resource‐limited. Emergency treatments are often performed under high‐stress conditions, involve non‐standard workflows, and may be covered by staff who have limited proficiency in performing non‐routine treatments due to rotating staff model. Physicians are frequently required to triage patients, delineate targets, and review plans concurrently, while physicists and therapists manage plan accuracy verification and treatment delivery. This time‐constrained convergence of responsibilities increases the risk of workflow inefficiencies, communication gaps, and treatment errors. RT emergency workflows would therefore benefit from the use of automation to compress and standardize planning and delivery processes to enhance treatment quality, accuracy, and safety. Development of a modified emergency workflow guided by dedicated in‐house software with a graphic‐user interface (GUI) was demonstrated to be advantageous for CBCT‐based emergency treatment;^14^ however, its use is currently limited to a single institution. While promising, the level of complexity of this in‐house development may hinder wide adaptation of this approach.

The purpose of this work is to describe the development, implementation, and early clinical experience of an alternative automated CT simulation‐free RT approach for emergent palliative treatment, with an emphasis on integrating ESAPI scripts within the treatment planning system (TPS) to enhance safety while maintaining minimal deviation from standard clinical processes. As other centers explore similar approaches, we believe that our experience can contribute to a broader discussion on the role of scripting and automation in improving the safety, reliability, and standardization of emergency RT care.

## 2 METHODS

### 2.1 Clinical setting

The workflow was designed to be used across multiple clinical sites in an academic center in a mixed‐vendor environment including a large number of CT simulators (GE and Siemens) and linear accelerators (Varian and Elekta), a common TPS and oncology information system (OIS) (Eclipse/ARIA v16.1), and an independent dose calculation tool (ClearCheck, Radformation). A team consisting of one physician, one medical resident, one qualified medical physicist (QMP), and two radiation therapists are assigned on a weekly rotating basis to cover potential emergent treatments.

### 2.2 Workflow design goals and requirements

The emergency treatment workflow was developed with two primary objectives: 1) to reduce the likelihood of human error by automating as many manual steps as practicable, and 2) to maintain close alignment with the institution's standard (non‐emergency) external beam radiation therapy (EBRT) workflow. Preserving familiarity with current non‐emergency clinical processes was considered essential to support safe and operationally efficient execution by on‐call clinical staff, especially since they may have to provide services at sites other than their home clinic during this time‐critical workflow.

### 2.3 Workflow evaluation for automation

The workflow was intentionally designed to leverage software platforms and clinical tools already in routine use across the institution. Since our department uses Varian TPS and OIS, ESAPI scripting was employed to automate as many steps as possible. Automation of the full workflow was limited by vendor restrictions within the OIS scripting environment. Specifically, some functionalities—such as scheduling—are not accessible through the current ESAPI interface.

**FIGURE 1:**
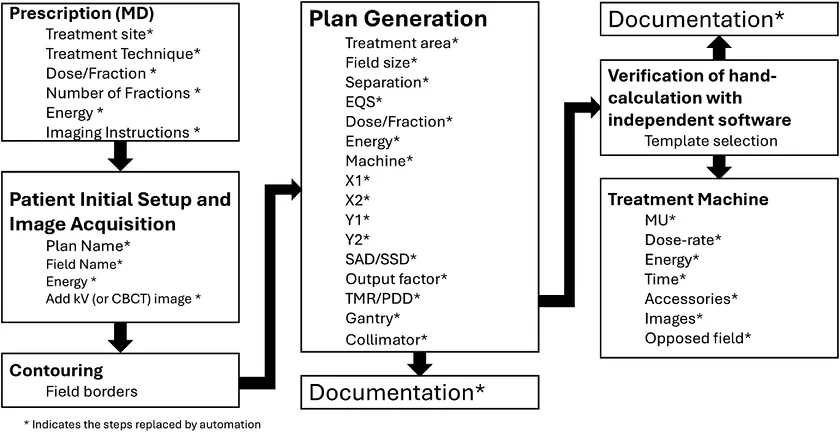
Overview of conventional emergency treatment workflow with steps replaced by automation.

Figure 1 demonstrates the critical steps in the conventional 2D emergency treatment workflow enumerating steps that were automated in the new 3D workflow. First, standardized emergency prescription templates specific to a treatment site were defined with default values (i.e., treatment site, technique, dose per fraction, number of fractions, beam energy and imaging instructions), while allowing physicians to modify as clinically indicated. Following image acquisition, pre‐planning data was automatically retrieved and prepared for the next step. These operations are well suited for automation via scripting within commercial TPS environments and align with previously published clinical implementations of scripting using vendor‐supported application programming interfaces.^4^, ^5^, ^6^, ^11^ In contrast, tasks requiring clinical interpretation or real‐time decision‐making were not automated. Manual steps performed by the physician included selection of correct prescription template, its modification if necessary and approval, treatment field definition and plan evaluation, which is consistent with published guidance emphasizing physician responsibility for target definition and plan review/approval.^9^, ^10^, ^17^

### 2.4 Script development

**FIGURE 2:**
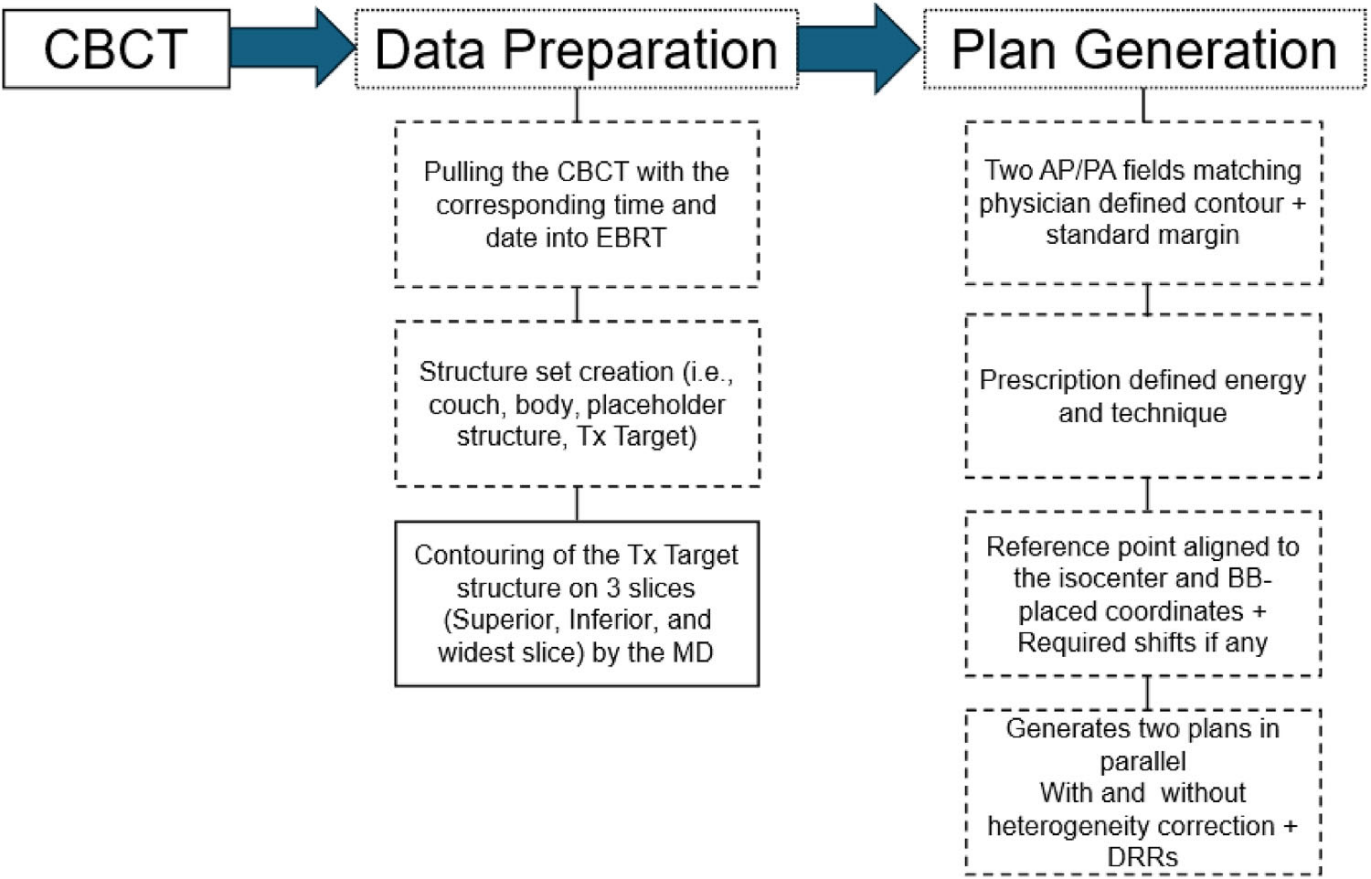
Overview of the CBCT‐based emergency radiotherapy workflow. Solid rectangles indicate manual clinical actions; dotted rectangles represent EmTxScript execution step. Dashed rectangles indicate actions performed automatically upon execution of EmTxScript.

ESAPI script (“EmTxScript”) execution was implemented as discrete, user‐initiated steps. This model allows verification of intermediate output results, such as the image dataset, prescription, and structure delineation, before proceeding to subsequent automated steps. Similar human‐in‐the‐loop strategies have been recommended to mitigate risk in the context of automated treatment planning and clinical scripting.^5^, ^11^, ^14^ Robust error handling and input validation were implemented to prevent execution under invalid conditions. For example, the script verifies that a prescription is present and properly associated with the selected course, and that any prescribed beam energy is supported by the treatment machine. Execution is halted if required data are missing, inconsistent, or clinically invalid. In addition, the user is guided through the clinical workflow with contextual prompts and feedback, highlighting missing or inconsistent selections, and providing direction for corrective action. Detailed descriptions of script logic, data flow, validation checks, and implementation specifics are provided in Section A of the supplementary material. In Figure 2, an overview of the scripted planning workflow is presented. This workflow is initiated by the physician immediately after the patient is positioned on the treatment couch and a CBCT is acquired.

As shown in Figure 2, there are two major steps in the workflow where EmTxScript execution is implemented: planning data preparation and plan generation. In planning data preparation, import and selection of the most recent CBCT is automated. Additional pertinent information is populated from the prescription. EmTxScript also automatically creates a structure set that includes the external contour (body), couch tops and a placeholder for a target structure (Tx_Target). There are no manual steps in this process except for verification that the correct CBCT dataset is selected in case of multiple CBCTs. In the developed workflow, the physician does not contour typical targets (e.g., GTV, CTV, PTV). Instead, the physician delineates the superior, inferior, and lateral extents of the treatment fields on three representative slices. EmTxScript is then executed a second time to generate the treatment field to cover the extent of the delineated structures in 3 dimensions. In the plan generation step, EmTxScript automatically creates AP/PA fields and an isocenter with respect to the Tx_Target center. If the treatment isocenter differs from the imaging isocenter or if a “merged” CBCT is acquired leading to an offset imaging isocenter, the script does account for these differences in the setup shifts provided to the users.

The EmTxScript generates two plans in parallel, with and without heterogeneity correction (HC) enabled. The default density‐correction algorithm, either AAA or Acuros XB, for each machine is utilized. While heterogeneity‐corrected dose calculation is expected to be more accurate on well‐calibrated CBCT images, this dual‐plan strategy allows comparison of isodose distributions and supports decision‐making when CBCT image quality or presence of anatomical implant raises concerns about the accuracy of tissue density representation. Internal checks raise warnings if the dose between the density‐corrected and uncorrected calculations differ by more than a user‐defined threshold (i.e., 5% in practice).

### 2.5 Survey design and analysis

#### 2.5.1 Survey development and distribution

To evaluate clinical perspectives on emergency treatment workflows and assess the perceived value of CBCT and automation, a brief survey was designed and administered before and after training on the script‐guided CBCT‐based workflow (3D workflow). The survey consisted of seven questions (see Supplement) designed to assess respondents’ comfort with performing emergency treatments using orthogonal MV imaging (2D workflow), identify challenges associated with emergency coverage, and determine which workflow steps were perceived as most difficult or error‑prone. Additional items evaluated perceptions of the 3D workflow, including benefits among those who supported its use and limitations/concerns among those who did not, as well as an overall assessment of workflow accuracy and operational efficiency. The post‐implementation survey repeated key items to enable direct comparison following training and initial clinical use of the automated 3D workflow.

Responses included Likert‑scale ratings of comfort and workflow performance, categorical evaluations of accuracy and efficiency, and multiple‑choice selections addressing perceived benefits and limitations. The survey was distributed to all clinical staff participating in emergency treatment coverage during the training sessions, and participation was voluntary. Responses were collected anonymously using Qualtrics (Qualtrics, Provo, UT).

#### 2.5.2 Analysis of survey data

Descriptive statistics were used to summarize the survey results. For analysis, responses on the 5‑point Likert scale were collapsed by combining categories on either side of the neutral response. Categorical variables were compared using the chi‑squared test. Statistical significance was assessed at the *p* < 0.05 level, and *p*‑values were adjusted (p‐adjusted) for multiple comparisons using the Benjamini–Hochberg (BH) false discovery rate (FDR) procedure.^18^

## 3 RESULTS

### 3.1 Clinical implementation of script‐based automation

**FIGURE 3:**
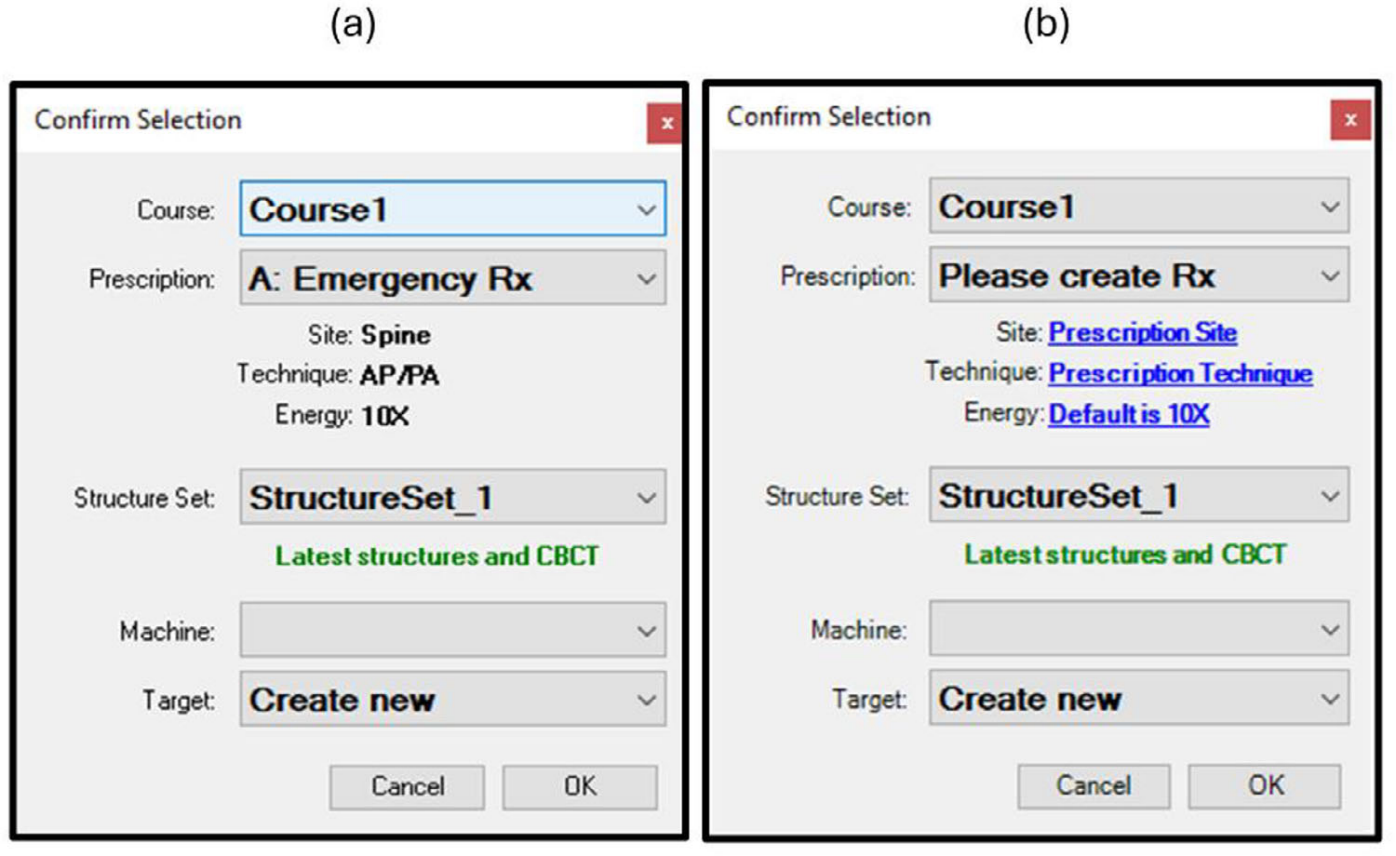
An example of the script interface with (a) and without (b) prescription.

Figure 3a demonstrates a screenshot of the EmTxScript interface, which was designed to be simple and intuitive. As shown in Figure 3a, the information that is pre‐processed by the script and displayed to the user for verification includes the parameters pulled from the latest prescription, the most recent image set, and auto‐generated structure set for contouring. The drop‐down options were designed to facilitate ease of modifications where necessary. To enhance the safety of this process, the warnings were designed to alert users when discrepancies were detected. For instance, as shown in Figure 3b, warning prompts are generated by EmTxScript if the prescription is missing the necessary elements (energy, site, fractionation) or if the structure set or target has incomplete segmentations.

### 3.2 Workflow efficiency and accuracy

EmTxScript was developed and soft launched to be used by a select group of users prior to full release across the multi‐site institution.

**FIGURE 4:**
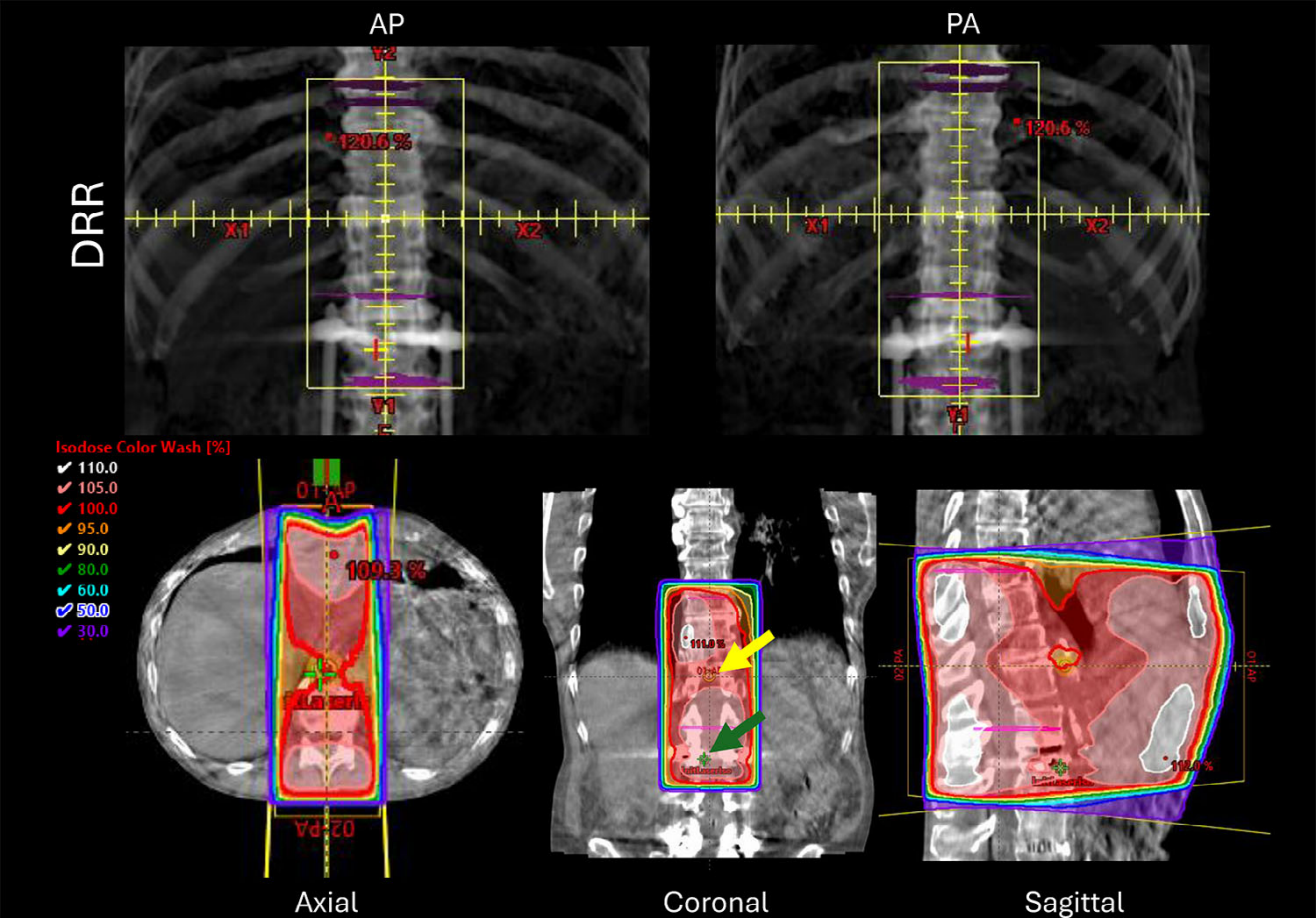
A sample clinical case treated with the automated CBCT‐based workflow: field borders on AP/PA DRRs and isodose distributions in color wash in axial, coronal, and sagittal planes. The imaging and treatment isocenters are shown by the green and yellow arrows, respectively.

Figure 4 shows digitally reconstructed radiographs (DRRs) with field borders matching the contoured surrogate target (pink) in both sup/inf and lateral directions and the dose distribution in axial, coronal, and sagittal slices for a clinical case. Plan generation and dose calculation were performed automatically with isocenter placement at the geometric center of the field and patient midline, equal beam weighting, heterogeneity corrections enabled. The dose distribution reflects a typical AP/PA treatment with symmetric fields and isocenter at the midline of the patient. The script also accounts for shifts from the initial imaging isocenter (e.g., the center of the first CBCT in a “merged” CBCT scenario) defined by in‐room lasers (green arrow in Figure 4) to the treatment isocenter at the midline (yellow arrow in Figure 4). The automatically calculated shifts can be verified by the physician and physicist during the plan check. The shifts are also included in the automatically uploaded treatment plan document and are readily available to the therapist during RT time‐out checks prior to the treatment. The auto‐generation of DRRs provides additional levels of safety.

We retrospectively compared 5 cases to evaluate the dose calculation accuracy. Table 1 shows a summary of five cases (10 Treatment fields) for which the dose calculation was retrospectively compared on CBCT and CT image sets. MUs for identical dose prescriptions calculated on CBCT datasets with enabled HC demonstrated close agreement with reference planning CT calculations (HC = ON) (Table 1). Across five clinical cases with both CT and CBCT acquisition that were recalculated using identical beam parameters and fractionation, the maximum monitor unit (MU) deviation between CT (HC = ON) and CBCT (HC = ON) was 2.2%. In comparison, disabling HC introduced larger discrepancies, with deviations up to 4.7%. Deformable registration of CBCT to CT was utilized to remove contribution from anatomical differences, therefore residual MU variations were attributable primarily to HU calibration differences.

**TABLE 1:** Percentage monitor unit (MU) differences across calculation methods with heterogeneity correction on (“HC = ON”) and off (“HC = OFF”). Values represent percentage difference per field (AP or PA beam).

Field	CT (HC = ON) vs CBCT (HC = ON)	CT (HC = ON) vs CBCT (HC = OFF)
1	0.0%	0.5%
2	0.5%	2.5%
3	2.2%	2.2%
4	0.0%	0.5%
5	0.5%	4.7%
6	1.9%	3.7%
7	0.5%	0.0%
8	0.0%	2.8%
9	0.5%	1.0%
10	1.1%	2.7%

### 3.3 Effectiveness of automation

The effectiveness of automation was evaluated by comparing the number of manual interactions required in the conventional emergency treatment workflow (2D workflow) with those required following implementation of the scripted, template‐driven process (3D workflow). Manual entries were identified for each step of the 2D emergency workflow, including prescription entry, plan configuration, beam setup, dose calculation, and plan parameters verification. Figure 1 shows eliminated manual data‐entry steps that were previously required in the 2D workflow, 35 entries in total. These reductions primarily occurred in prescription configuration, plan generation, loading the patient's plan on the treatment machine at the time of the treatment, and documentation‐related steps. By replacing manual entries with deterministic script‐driven actions, the automated 3D workflow reduced opportunities for transcription errors and configuration inconsistencies while simplifying execution during time‐constrained emergency scenarios.

To evaluate the operational efficiency, time metrics were collected for patients who were treated with this newly developed workflow. Table 2 shows time intervals collected from Offline Review for the first 5 patients treated with automated workflow. The intervals between the CBCT and the treatment initiation were calculated. The overall treatment preparation time on average was found to be just under 50 min. Details of the data extraction are presented in Figure B1 in the supplementary material. Shorter times were observed for spine treatments, while the longest time occurred when treating a lateral rib. These times do not include time to position the patient and finalize the paperwork after treatments, since these processes were not modified. Table B1 shows the breakdown of cumulative time from patient entering the treatment room to patient exiting the treatment room after the treatment for two sample cases.

**TABLE 2:** Time from CBCT acquisition to treatment initiation for five emergency cases.

Case	Treatment site	Time from CBCT to treatment initiation (min)
1	C‐Spine	44:01
2	T‐Spine	41:20
3	L‐Spine	43:54
4	Left Rib	67:40
5	Rectum	51:39
**Average**		**49:54**

### 3.4 Survey outcomes

The pre‐training and post‐training surveys were completed by 63 and 36 staff, respectively. Fewer participants completed the post‐training survey due to limited time and availability.

### 3.5 Comparison of the 2D and 3D imaging‐based treatment workflows

There was a statistically significant increase in comfort (somewhat and extremely comfortable) with the CBCT‑based procedure (3D) relative to the MV port‑film–based approach (2D) (*p* < 0.001), with reported comfort rising from 28% to 69% (Figure S1). After collapsing the rating categories related to accuracy and efficiency, the 2D procedure was predominantly judged as poor or very poor, whereas the CBCT‑based 3D procedure was predominantly rated as good or excellent, indicating a markedly stronger preference among respondents for the CBCT‑based approach (*p* < 0.001). (Figure S2). When asked about the most challenging step in the procedure, no statistically significant differences were observed between procedure types although “performing hand calculation” showed the largest numerical reduction from 47% to 28% for the CBCT‐based procedure (Figure S3). It is important to note that “performing hand calculation” refers to the MUs calculated either manually via lookup tables in the 2D workflow or via the script in the 3D workflow. Hence, the reduction in perceived challenge due to “performing hand calculation” was anticipated as this step was fully automated in the 3D workflow.

### 3.6 Comparison of perceptions pre‐ and post‐training

Regarding whether CBCT could improve emergency treatments, 93% of respondents answered positively before training, and this increased to 100% after training. (Figure S4) Across all categories of perceived benefits of the CBCT‐based procedure, changes from pre‑ to post‑training were modest (< 3%), except for the proportion selecting “faster treatments” which increased from 20% to 31%. The most frequently selected benefit at both time points was “safer treatments” (37% pre‑training and 33% post‑training). (Figure S5) Overall, respondents reported significant shifts in two perceived challenges between the two procedure types. The proportion identifying “unclear procedure” as a top challenge decreased significantly for the CBCT‑based approach (p_adjusted < 0.012), whereas endorsements of “patient comfort” as a challenge increased significantly for the CBCT‑based approach (p_adjusted < 0.016). All other challenge categories showed no statistically significant differences between the two procedures as shown in Figure S6.

## 4 DISCUSSION

This study details the creation, execution, and preliminary clinical evaluation of an automated simulation‐free CBCT‐based RT workflow for urgent palliative care. While various simulation free workflows have been implemented by others,^4^, ^5^, ^6^, ^11^, ^14^ our approach was developed with a focus on scripting to maximize automation while maintaining consistency with routine clinical practice and established team roles.

### 4.1 Safety and efficiency

Time metrics collected for patients treated using this workflow demonstrated that the full emergency treatment process, from the time the patient enters the treatment room to the time the patient leaves the room, could be completed in less than 1.5 h, consistent with institutional expectations and previously reported benchmarks for expedited palliative RT workflow.^4^, ^8^, ^14^, ^19^ This overall time includes steps that cannot be automated and are difficult to expedite at this time, such as positioning the patient on the treatment couch. On the other hand, the steps that included automation, mainly related to plan generation, were found to be completed within 50 min, on average. The ability to complete the plan preparation process in under 1 h reflects the advantages of automation in streamlining multiple steps within the workflow. For instance, a simple and intuitive interface with appropriate prompts, as shown by an example in Figure 3b, contributes to operational efficiency by appropriately guiding the user. The overall emergency process was designed to align with existing clinical workflow and personnel responsibilities to ease adaptability and maintain operational efficiency with fewer resources. For example, since dosimetrists are not part of our emergency treatment workflow, automation via scripting enabled treatment planning while tasks such as secondary MU calculations and treatment plan documentation were automated via a commercially available solution (i.e. Radformation) enabling them to be overseen by the on‐call team. Although time estimates were not collected for the legacy 2D workflow, we estimate the times to be in the order of a few hours, in agreement with previously reported results.^14^ Therefore, the proposed workflow demonstrates the feasibility of achieving safety without compromising efficiency, overcoming a major limitation of the original 2D workflow.

To estimate the operational efficiency indirectly, we highlighted the number of manual entries in Figure 1, which already would increase the total time significantly. It is important to note that each one of the steps listed under plan generation in Figure 1 would have to be repeated manually every time physician would like to change the treatment field after examining the port films. One challenge faced by large academic institutions with multiple satellite locations is maintaining effective communication and coordination across teams at different sites. To minimize treatment delays due to lack of communication and coordination, an on‐call contact sheet is generated and emailed weekly to the on‐call team while providing access to the emergency procedure in a centralized location.

### 4.2 Dose calculation accuracy

An important consideration when using CBCT for treatment planning is related to accuracy of dose calculations. This was evaluated by a direct comparison of treatment plans generated from CT simulation and CBCT images with HC, which demonstrated agreement within 3%, consistent with a previous study indicating that MUs calculated based on the dose at a prescription point differed by no more than 5%.^19^ Internal checks raise warnings if the dose between the density‐corrected and uncorrected calculations differ by more than a user‐defined threshold (i.e., 5% in practice). If the discrepancy is found, the QMP would need to identify the root cause of disagreement (e.g., an imaging artifact) and select a plan for treatment. A non‐heterogeneity corrected plan may be used in some situations, particularly if the beam pathway does not intersect lung tissue or metal.

### 4.3 Staff perception of automated workflow

The survey results demonstrated strong institutional readiness and clinical support for transitioning to the CBCT‐based emergency RT workflow. While the majority of clinical staff lacked confidence in the existing emergency process, particularly with respect to manual dose calculations based on MV port films, the survey noted reduction in the challenge of “performing hand calculations” (47% to 28%) with the CBCT‐based workflow. An increased comfort level in performing a CBCT‐based procedure may be attributed to the inherent advantage of 3D visualization over MV port films and better alignment of the emergency process to routine clinical workflows, which are primarily 3D‐based. Reported comfort and confidence also improved substantially following training and early clinical exposure, with workflow ratings shifting from predominantly poor or fair to good or excellent. In addition, the significant reduction in “unclear procedure” after training shows the effectiveness of accompanying training/education. Overall, the perception of CBCT‐based procedure as ‘a safer and faster treatment’ by the staff suggests that automation was viewed as a positive implementation.

### 4.4 Adaptability

Although development of an in‐house application for CBCT‐based planning for palliative RT on a conventional linear accelerator has been described previously by Tegtmeier et al,^14^ this approach relies on a graphic user interface and requires significant programming effort that may hinder its widespread use across institutions without the infrastructure needed to support such software development. In addition, the workflow assigns the plan generation task to the therapist. In contrast, our work presents an alternative workflow design focusing primarily on automating the current treatment planning process through scripting within the TPS. This approach limits the need for educating staff on a new interface, while also preserving the therapists’ role in a manner consistent with standard clinical procedures where they are responsible for imaging and treatment but not plan generation. An additional simplification in our workflow was achieved by requiring target delineation on two or three slices rather than the entire volume as necessitated by the workflow described by Tegtmeier et al. Thus, our work offers an alternative method for implementing a CBCT‐based scripting‐driven palliative workflow that is less programming intensive and more closely aligned with our institutional clinical practice.

Another unique feature of the EmTxScript is its adaptability across heterogeneous treatment platforms. Although Varian and Elekta linear accelerators differ substantially in jaw design, multi‐leaf collimator configuration, and field‐shaping mechanics, Elekta‐specific constraints are handled internally within the EmTxScript framework while maintaining identical script execution steps, which was a key requirement for this implementation. This design minimizes training requirements, reduces the risk of user error when operating across multiple platforms, and supports scalability within large, multi‐center institutions. The ability to accommodate Elekta‐specific constraints—such as Y‐only jaw motion and MLC‐defined field shaping—without altering the external workflow underscores the flexibility of scripting‐based solutions in managing hardware heterogeneity. Finally, the EmTxScript is also well suited for emergent cases during clinical hours, as it helps overcome resource limitations such as an overscheduled CT simulator and/or limited dosimetry staff.

One motivating factor for proposing this expedited workflow is to reduce the overall time from simulation to treatment given that the patient's comfort and compliance could be a challenge for palliative treatments. Although it is not necessary to remove the patient from the couch between the CBCT acquisition and treatment delivery, it is feasible to do so and to reposition the patient using the initial laser marks.

### 4.5 Limitations

Several limitations are associated with the use of scripted automation of the emergency workflow. A key limitation of the proposed workflow is that, in the event of script failure, users would be unable to generate a treatment plan using the automated process and would need to resort to a conventional 2D workflow. While the risk of script failure was deemed low due to its robustness during testing, periodic end‐to‐end script validation was implemented to further mitigate this risk. Furthermore, the main concern is that with broader adoption of the 3D workflow, the 2D fallback process may become progressively less familiar to staff, potentially increasing risk through unintended deskilling. To address this concern, educational videos for both the 2D and 3D workflows were created and placed in a departmental repository for team members to review prior to their on‐call assignments. While it may also be feasible to use the available 3D CBCT for treatment planning in the event of script failure, this pathway is currently not supported at our institution since the dosimetrists are not part of the weekend emergency treatment team. As a result, an inexperienced treatment planner would have to manually plan the emergency treatment, which would increase the failure modes. For cases with extended scan requirements and treatment platforms without the capability of merging multiple CBCTs, the CT simulation must be performed. Nonetheless, the script was designed to function in a similar fashion to that of CBCT‐simulation to maintain consistency.

The survey was carried out before and after training on the CBCT‑based workflow. As participation was voluntary, not all individuals trained completed the survey, introducing the possibility of selection bias. In addition, responses may have been influenced by the department's stated intention to transition toward a 3D‑based workflow, as this intent was not blinded. Nonetheless, the survey findings aligned with initial informal feedback from clinical leaders, who reported that staff were uneasy with the 2D‑based workflow because it was infrequently used and perceived to be more prone to manual errors. For example, staff members noted that they do not routinely perform manual monitor unit calculations and identified this step as the greatest potential source of error. Another limitation relates to the survey design itself. The predefined response options may not have captured all potential error modes or challenges encountered in clinical practice. Furthermore, respondents were required to select a limited number of challenges, making it difficult to gauge the relative importance of each issue. Despite these limitations, the survey identified two challenges that differed significantly between workflows: “unclear procedure” was selected more frequently for the 2D workflow, whereas “patient comfort” was selected more frequently for the 3D workflow. These findings are consistent with departmental observations that staff are less familiar with the rarely used 2D workflow, and that patients are expected to remain on the treatment couch longer during 3D workflow planning as the script generates the treatment plan. This concordance between survey results and observed practice increased confidence in the validity of the findings.

### 4.6 Future directions

Creating a simple, intuitive, and highly adaptable script was a critical component as it not only reduced a large number of manual steps but also allowed its seamless integration into the proposed CBCT‐based workflow. While the current implementation focuses on CBCT‐based emergency treatments, the underlying automation framework is largely agnostic to the source of three‐dimensional imaging, with the primary distinction arising in setup shift calculation. This flexibility positions the approach as a generalizable model for emergency and expedited RT workflows as institutions continue to explore simulation‐free and automation‐driven care pathways.^10^, ^11^, ^13^, ^14^ Future work will also focus on evolving the EmTxScript to support MLC‐shaped treatments, same‐day VMAT treatments ^20^ as well as adaptive RT.^21^ The potential of auto‐contouring spine targets will also be investigated. With successful clinical implementation of the current approach, our next step is to perform a failure mode and effects analysis (FMEA) to understand the risk modes associated with the proposed workflow and further improve the accuracy and safety of emergency treatments.

## 5 CONCLUSIONS

This work demonstrates the potential of TPS scripting to meaningfully compress and standardize emergency RT workflows while preserving essential clinical oversight. By automating deterministic tasks, including plan creation, beam configuration, dose calculation, and setup preparation, the workflow reduces reliance on manual data entry and repetitive configuration steps that are prone to error under time pressure. At the same time, tasks requiring clinical judgment, such as target definition and image verification, remain explicitly manual, consistent with recommended human‐in‐the‐loop automation principles. The observed reduction in manual interactions, combined with treatment preparation times consistently under 1.5 h, suggests that scripting can enhance safety and operational efficiency without disrupting established clinical safeguards.

## AUTHOR CONTRIBUTIONS

All authors contributed to the methodology development and the manuscript preparation, final editing, and review. Data acquisition, analysis and interpretation: Shadab Momin, Eduard Schreibmann, Justin Roper, Hania Al‐Hallaq, Yulia Lyatskaya. Statistical analysis: Hania Al‐Hallaq Supervision: Eduard Schreibmann, Hania Al‐Hallaq, Yulia Lyatskaya.

## CONFLICT OF INTEREST STATEMENT

Hania Al‐Hallaq—Serves as Chair of a consortium on the Halcyon platform sponsored by Varian Medical Systems.

## DATA ACCESSIBILITY STATEMENT

Available upon request.

## ETHICS STATEMENT

IRB was not required for this study.

## DISCLOSE USE OF AI

AI tools (Microsoft Copilot and ChatGPT) were used to assist with drafting and editing the manuscript for clarity and conciseness. All content was reviewed by the authors, who take full responsibility for the final text.

## Supporting information



Supporting information: acm270814‐sup‐0001‐SuppMat1.docx

Supporting information: acm270814‐sup‐0002‐SuppMat2.pdf

Supporting information: acm270814‐sup‐0003‐SuppMat3.pdf

Supporting information: acm270814‐sup‐0004‐SuppMat4.pdf

Supporting information: acm270814‐sup‐0005‐SuppMat5.pdf

Supporting information: acm270814‐sup‐0006‐SuppMat6.pdf

Supporting information: acm270814‐sup‐0007‐SuppMat7.pdf

Supporting information: acm270814‐sup‐0008‐SuppMat8.pdf
