# Supplementary material for: An institutional experience of a scripting‐driven automation of the emergent palliative radiotherapy workflow
Source: J Appl Clin Med Phys. 2026 Sep 23;27(10):e70814. doi: 10.1002/acm2.70814 (PMC13598930; doi:10.1002/acm2.70814)
Supplement: Supplementary file 1 [file ACM2-27-e70814-s001.pdf]

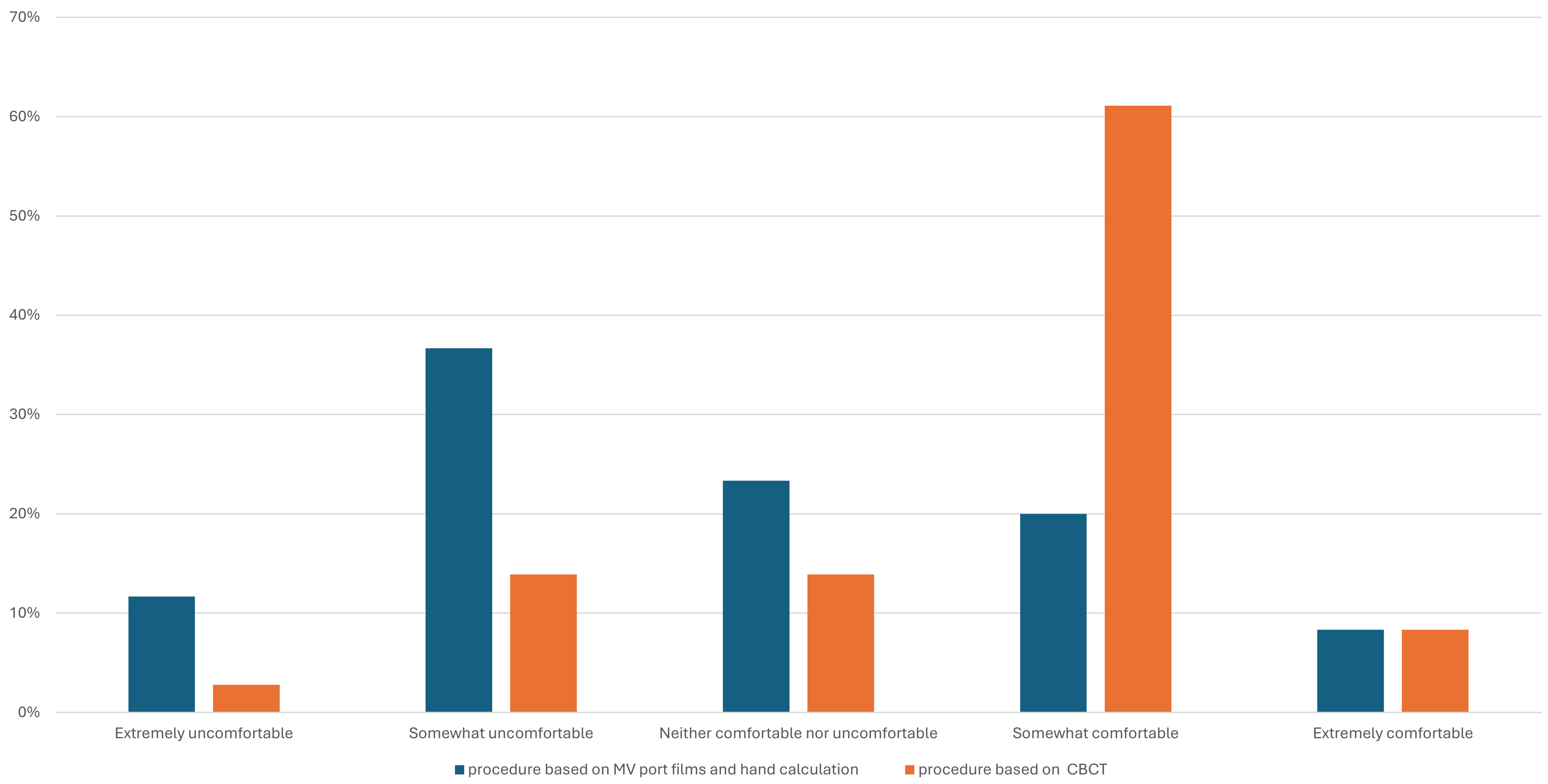

70%
60%
50%
40%
30%
20%
10%
0%
Extremely uncomfortable
Somewhat uncomfortable
Neither comfortable nor uncomfortable
Somewhat comfortable
Extremely comfortable
procedure based on MV port films and hand calculation
procedure based on CBCT
