# Supplementary material for: An institutional experience of a scripting‐driven automation of the emergent palliative radiotherapy workflow
Source: J Appl Clin Med Phys. 2026 Sep 23;27(10):e70814. doi: 10.1002/acm2.70814 (PMC13598930; doi:10.1002/acm2.70814)
Supplement: Supplementary file 2 [file ACM2-27-e70814-s002.pdf]

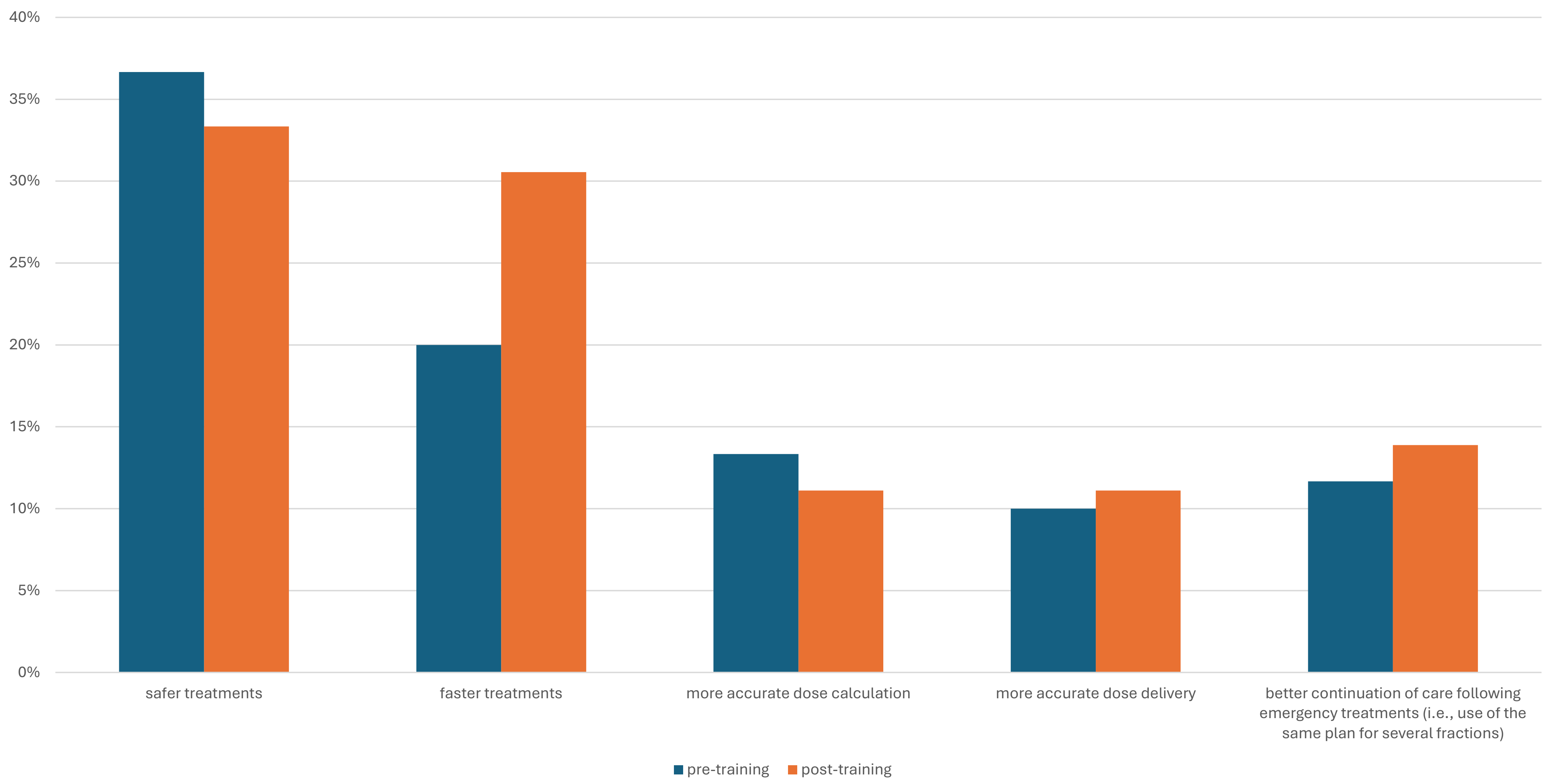

40%
35%
30%
25%
20%
15%
10%
5%
0%
safer treatments
faster treatments
more accurate dose calculation
more accurate dose delivery
better continuation of care following emergency treatments (i.e., use of the same plan for several fractions)
pre-training
post-training
