# Supplementary material for: An institutional experience of a scripting‐driven automation of the emergent palliative radiotherapy workflow
Source: J Appl Clin Med Phys. 2026 Sep 23;27(10):e70814. doi: 10.1002/acm2.70814 (PMC13598930; doi:10.1002/acm2.70814)
Supplement: Supplementary file 3 [file ACM2-27-e70814-s003.pdf]

(a)

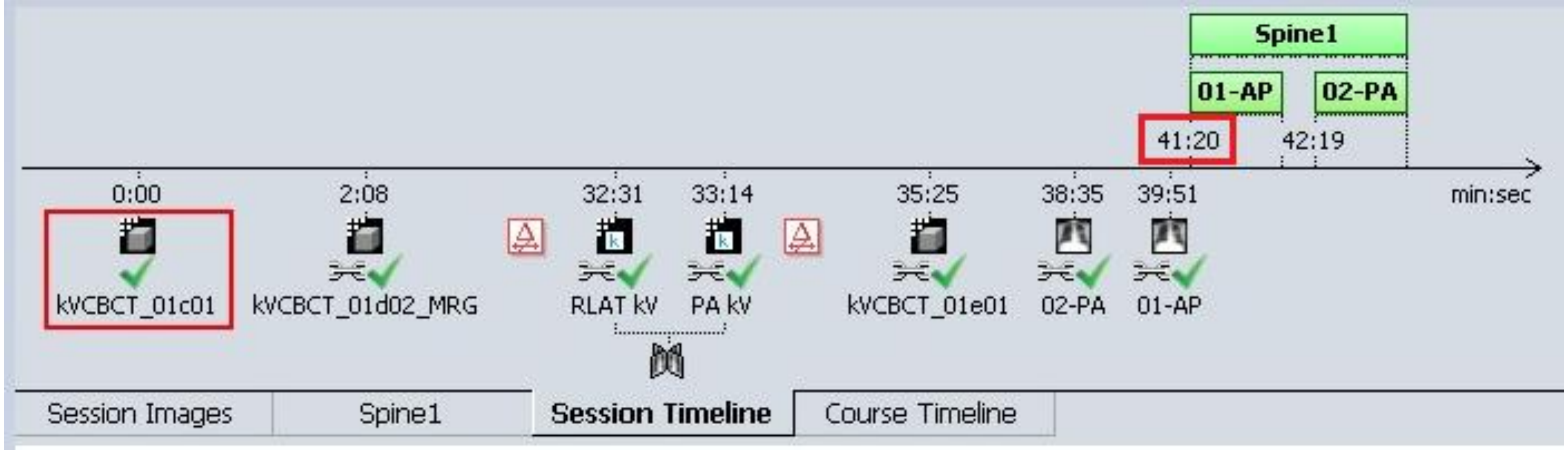

Spine1
01-AP
02-PA
41:20
42:19
0:00
2:08
32:31
33:14
35:25
38:35
39:51
min:sec
kVCBCT_01c01
kVCBCT_01d02_MRG
RLAT kV
PA kV
kVCBCT_01e01
02-PA
01-AP
Session Images
Spine1
Session Timeline
Course Timeline


(b)

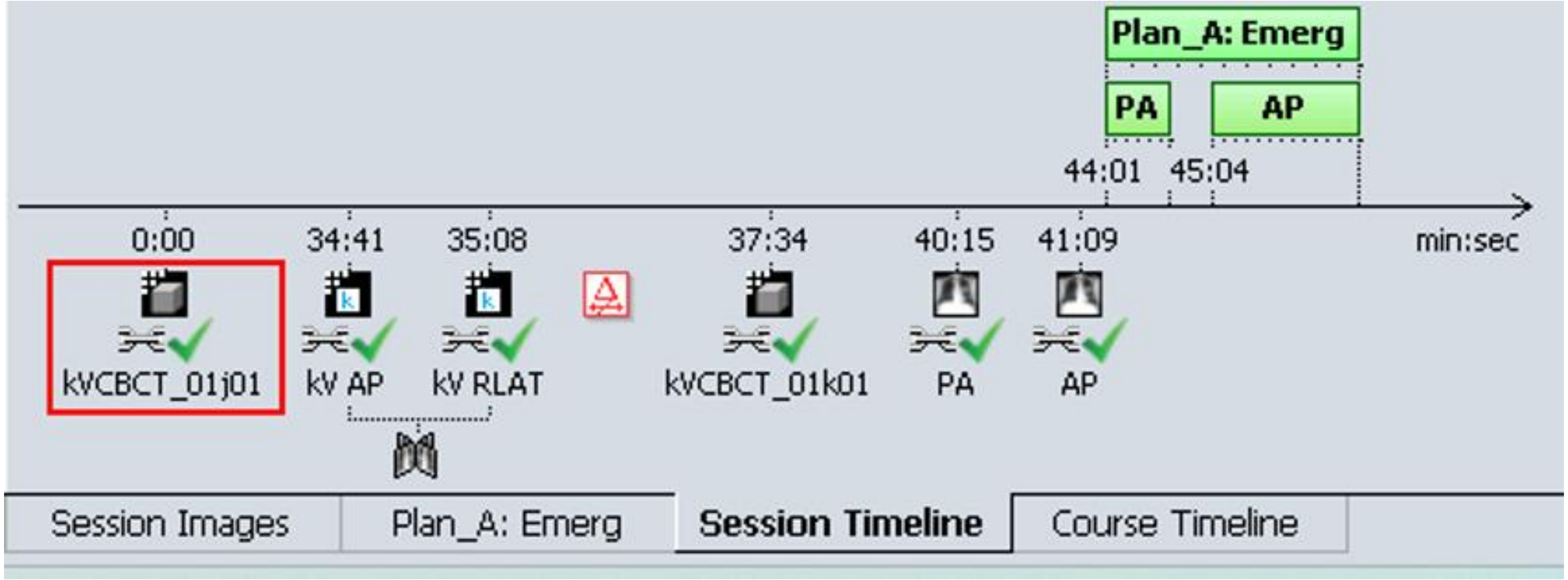

Plan_A: Emerg
PA
AP
44:01
45:04
0:00
34:41
35:08
37:34
40:15
41:09
min:sec
kVCBCT_01j01
kV AP
kV RLAT
kVCBCT_01k01
PA
AP
Session Images
Plan_A: Emerg
Session Timeline
Course Timeline
