# Supplementary material for: An institutional experience of a scripting‐driven automation of the emergent palliative radiotherapy workflow
Source: J Appl Clin Med Phys. 2026 Sep 23;27(10):e70814. doi: 10.1002/acm2.70814 (PMC13598930; doi:10.1002/acm2.70814)
Supplement: Supplementary file 4 [file ACM2-27-e70814-s005.pdf]

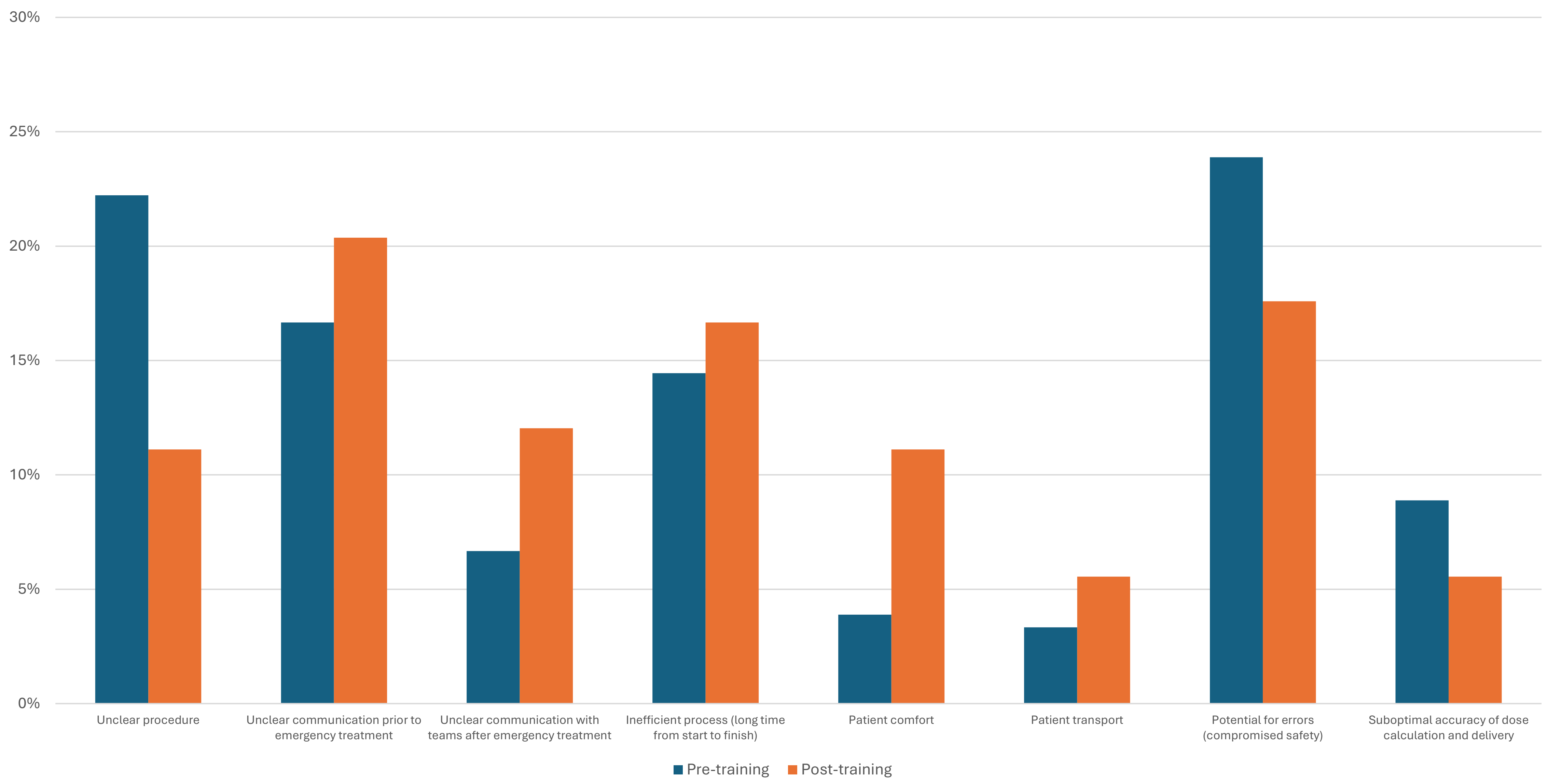
30%
25%
20%
15%
10%
5%
0%
Unclear procedure
Unclear communication prior to emergency treatment
Unclear communication with teams after emergency treatment
Inefficient process (long time from start to finish)
Patient comfort
Patient transport
Potential for errors (compromised safety)
Suboptimal accuracy of dose calculation and delivery
Pre-training
Post-training
