# Supplementary material for: An institutional experience of a scripting‐driven automation of the emergent palliative radiotherapy workflow
Source: J Appl Clin Med Phys. 2026 Sep 23;27(10):e70814. doi: 10.1002/acm2.70814 (PMC13598930; doi:10.1002/acm2.70814)
Supplement: Supplementary file 5 [file ACM2-27-e70814-s006.pdf]

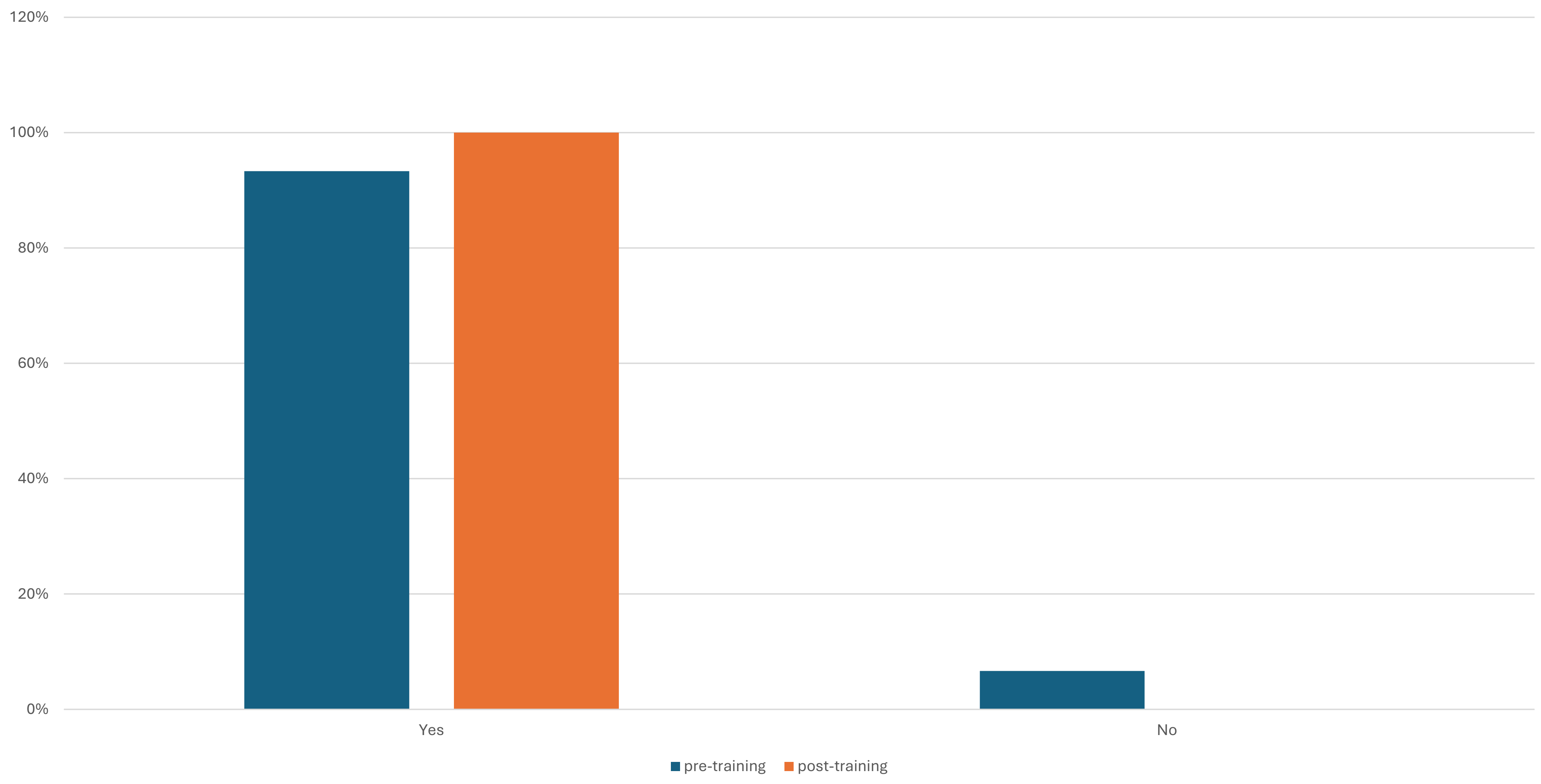
120%
100%
80%
60%
40%
20%
0%
Yes
No
pre-training
post-training
