# Supplementary material for: An institutional experience of a scripting‐driven automation of the emergent palliative radiotherapy workflow
Source: J Appl Clin Med Phys. 2026 Sep 23;27(10):e70814. doi: 10.1002/acm2.70814 (PMC13598930; doi:10.1002/acm2.70814)
Supplement: Supplementary file 7 [file ACM2-27-e70814-s008.pdf]

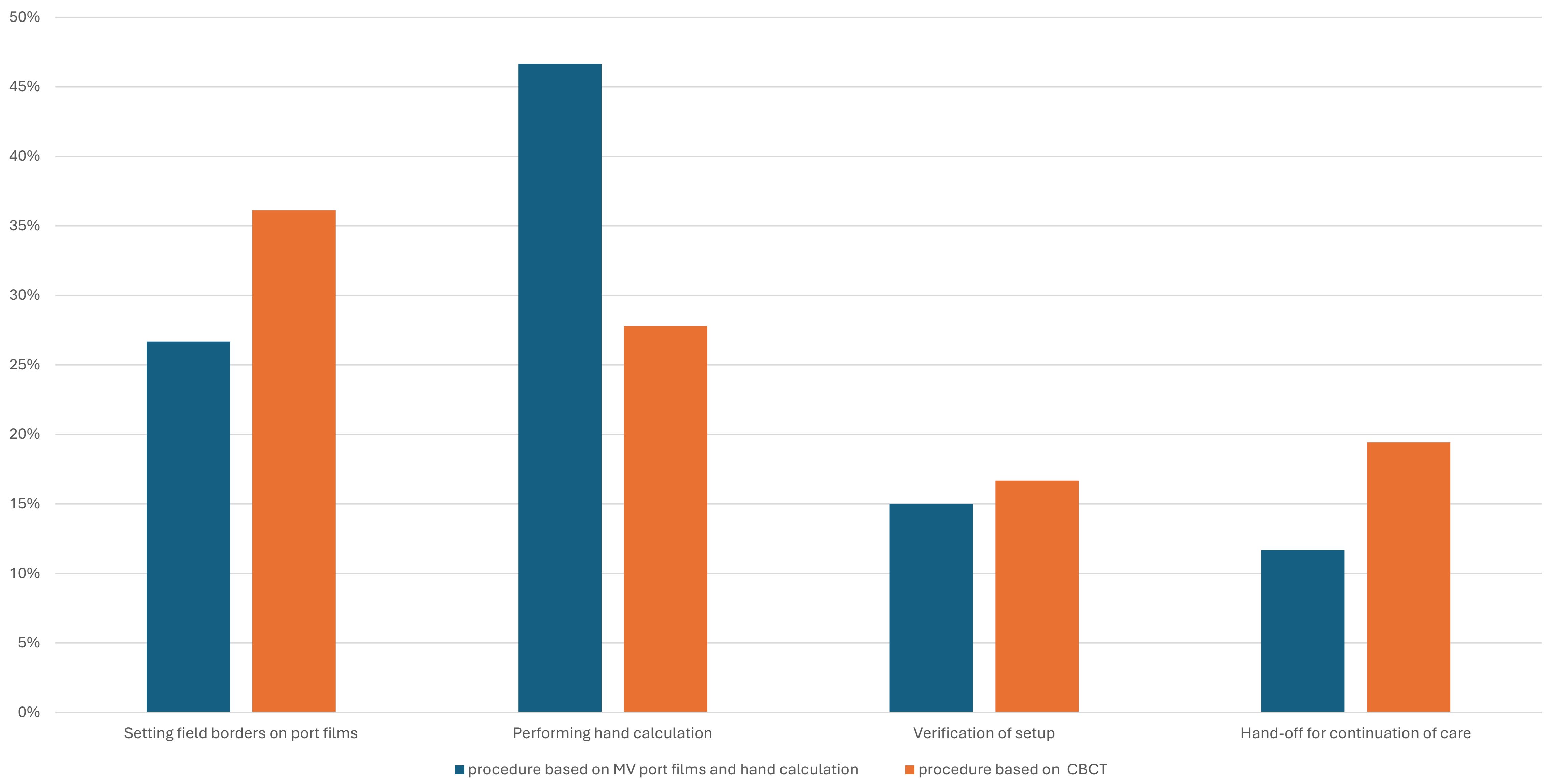
50%
45%
40%
35%
30%
25%
20%
15%
10%
5%
0%
Setting field borders on port films
Performing hand calculation
Verification of setup
Hand-off for continuation of care
procedure based on MV port films and hand calculation
procedure based on CBCT
