# Supplementary material for: An institutional experience of a scripting‐driven automation of the emergent palliative radiotherapy workflow
Source: J Appl Clin Med Phys. 2026 Sep 23;27(10):e70814. doi: 10.1002/acm2.70814 (PMC13598930; doi:10.1002/acm2.70814)
Supplement: Supplementary file 8 [file ACM2-27-e70814-s004.docx]

**Supplementary Material**

A. **Code and Clinical Step Mapping**

Figure 2 presents a schematic overview of EmTxScript in the CBCT-based emergency radiotherapy workflow. Below is the detailed explanation of each step from CBCT acquisition to plan generated by EmTxScript. This section also includes a brief explanation of platform-specific implementation of EmTxScript.

**1. CBCT Acquisition (Manual; Figure 2)**

As indicated in Figure 2, the workflow begins with acquisition of a cone-beam CT (CBCT) or extended CBCT dataset at the treatment console. This step is performed manually by radiation therapists using standard imaging protocols appropriate for the treatment site. Adequate anatomical coverage and image quality are confirmed prior to proceeding, as volumetric imaging serves as the foundation for all subsequent scripted steps.

**2. Data Preparation and Image Handling (Scripted; Figure 2)**

Following CBCT acquisition, the ESAPI script automatically performs data preparation tasks (Figure 2). These include identification of the most recent volumetric image set, creation of a structure set if one does not already exist, assignment of a default scanner model, and extraction or generation of a body contour. These steps standardize the dataset and ensure compatibility with dose calculation algorithms, eliminating variability associated with manual image preparation.

**3. Structure Set Initialization and Target Definition (Scripted + Manual; Figure 2)**

As indicated in data preparation stage in Figure 2, the script inserts a placeholder treatment structure into the structure set to guide physician input. The physician then manually defines the superior–inferior and lateral extent of the treatment volume on a limited number of axial slices. Full volumetric contouring is intentionally avoided to minimize time burden. The script verifies that the target structure is non-empty before proceeding, preventing plan generation from incomplete or invalid data.

**4. Prescription and Technique Validation (Templated and Scripted Checks; Figure 2)**

Prescription parameters—including treatment site, technique, energy, and fractionation—are automated via standardized emergency prescription templates). Prior to plan generation, the script validates prescription parameters to ensure they fall within supported emergency use cases (e.g., spine or brain treatments, AP/PA or opposed lateral techniques, and acceptable fractionation ranges). These validation checks serve as guardrails that prevent unsupported or potentially unsafe configurations (Figure 3).

**5. Automated Plan Generation and Beam Geometry (Scripted; Figure 2)**

Once target and prescription information are validated, the script generates the treatment plan automatically (Figure 2). While the treatment isocenter is placed centrally within the delineated treatment field and aligned with the patient’s midline, the imaging isocenter is defined by the center of the initial CBCT and is marked based on the lasers at the time of setup. Since the imaging isocenter remains centered on the first acquired CBCT when multiple CBCT are merged, the script extracts the imaging isocenter and calculates the translational 3D shifts to the treatment isocenter. For spine treatments, opposed AP/PA beams are created and fitted to the target extent. For brain treatments, opposed lateral beams are generated. Beam naming, reference point creation, and imaging field insertion are standardized programmatically to reduce variability and facilitate review.

**6. Dose Calculation, Normalization, and Safety Evaluation (Scripted; Figure 2)**

As indicated in Figure 2, dose calculation is performed automatically with heterogeneity correction enabled, and the plan is normalized at the isocenter. To evaluate sensitivity to CBCT HU variability and tissue heterogeneity, the script generates a secondary plan copy with heterogeneity correction disabled. This dual-calculation approach provides an additional safety check without delaying treatment.

**7. Platform-Specific Implementation: Varian and Elekta Systems (Scripted)**

On Varian linear accelerators, beam apertures are primarily defined using symmetric jaw motion. On Elekta systems, which employ Y-only jaws, the script derives multileaf collimator (MLC) leaf openings in the X-direction based on jaw-fitted target extents. Despite these internal differences, the execution sequence and user interaction remain identical across platforms.

**8. Plan Review, Approval, and Treatment Delivery** Final plan review, physics approval, and treatment authorization are performed manually according to institutional emergency procedures. Standard imaging verification is used prior to delivery. By design, automation supports—but does not replace—clinical judgment at this stage, ensuring that responsibility for treatment delivery remains within established clinical roles.

B. **Summary of time intervals**

Figure B1: Examples of time intervals extracted from Offline Review from the first CBCT acquisition to treatment initiation for two representative cases.

**Table B1:** Summary of the cumulative time (hh:mm) from patient entering the treatment room from start to patient leaving the treatment room after the treatment for two sample cases.

| **Major steps in emergency treatment** | **Pt.1 (hh:mm)** | **Pt.2 (hh:mm)** |
| --- | --- | --- |
| Room prep | 0:00 | 0:00 |
| Patients enter treatment room | 0:01 | 0:00 |
| First CBCT by the therapist and hands off to the MD | 0:08 | 0:05 |
| MD completes tasks and hands off to the QMP | 0:26 | 0:21 |
| QMP completes tasks and hands off to the therapists for treatment | 0:35 | 0:37 |
| Verification imaging (CBCT or kV/kV) | 0:40 | 0:43 |
| Beam On | 0:48 | 0:49 |
| Treatment complete | 0:50 | 0:51 |
| Patient leaves treatment room | 0:55 | 0:56 |
| **Total time from the beginning to end:** | **55min** | **56min** |

**Supplementary Figures**

|  | procedure based on MV port films and hand calculation | procedure based on CBCT |
| --- | --- | --- |
| Extremely uncomfortable | 11.6667% | 2.77778% |
| Somewhat uncomfortable | 36.6667% | 13.8889% |
| Neither comfortable nor uncomfortable | 23.3333% | 13.8889% |
| Somewhat comfortable | 20% | 61.1111% |
| Extremely comfortable | 8.33333% | 8.33333% |

**Figure S1:** Comparison of participant-reported comfort levels in covering emergency treatments using procedures based on MV portal films with hand calculations versus CBCT-based procedures.

|  | procedure based on MV port films and hand calculation | procedure based on CBCT |
| --- | --- | --- |
| Very Poor | 1.66667% | 0% |
| Poor | 33.3333% | 0% |
| Fair | 46.6667% | 16.6667% |
| Good | 15% | 61.1111% |
| Excellent | 1.66667% | 22.2222% |

**Figure S2.** Participant-reported ratings of overall accuracy and efficiency of emergency treatment procedures, comparing methods based on MV portal films with hand calculations versus CBCT-based procedure

|  | procedure based on MV port films and hand calculation | procedure based on CBCT |
| --- | --- | --- |
| Setting field borders on port films | 26.6667% | 36.1111% |
| Performing hand calculation | 46.6667% | 27.7778% |
| Verification of setup | 15% | 16.6667% |
| Hand-off for continuation of care | 11.6667% | 19.4444% |

**Figure S3.** Participant-identified most challenging steps in covering emergency treatment procedures, comparing workflows based on MV portal films with hand calculations and CBCT-based procedures.

|  | pre-training | post-training |
| --- | --- | --- |
| Yes | 93.3333% | 100% |
| No | 6.66667% | 0% |

**Figure S4.** Percentage of respondents indicating whether utilization of CBCT could provide improvements in emergency treatment coverage, comparing pre‑training and post‑training survey responses.

|  | pre-training | post-training |
| --- | --- | --- |
| safer treatments | 36.6667% | 33.3333% |
| faster treatments | 20% | 30.5556% |
| more accurate dose calculation | 13.3333% | 11.1111% |
| more accurate dose delivery | 10% | 11.1111% |
| better continuation of care following emergency treatments (i.e., use of the same plan for several fractions) | 11.6667% | 13.8889% |

**Figure S5.** Pre‑training and post‑training comparison of respondent perceptions regarding which aspects of emergency treatment workflows benefit most from a CBCT‑based procedure, including treatment safety, efficiency, dose calculation accuracy, dose delivery accuracy, and continuity of care.

|  | Pre-training | Post-training |
| --- | --- | --- |
| Unclear procedure | 22.2222% | 11.1111% |
| Unclear communication prior to emergency treatment | 16.6667% | 20.3704% |
| Unclear communication with teams after emergency treatment | 6.66667% | 12.037% |
| Inefficient process (long time from start to finish) | 14.4444% | 16.6667% |
| Patient comfort | 3.88889% | 11.1111% |
| Patient transport | 3.33333% | 5.55556% |
| Potential for errors (compromised safety) | 23.8889% | 17.5926% |
| Suboptimal accuracy of dose calculation and delivery | 8.88889% | 5.55556% |

**Figure S6.** Comparison of the three most frequently reported challenges encountered when covering emergency procedures, as identified by participants before and after training, including procedural clarity, communication, efficiency, patient-related factors, safety concerns, and dose calculation accuracy.
